# Guillain‐Barré syndrome: History, pathogenesis, treatment, and future directions

**DOI:** 10.1111/ene.16346

**Published:** 2024-05-16

**Authors:** Richard A. C. Hughes

**Affiliations:** ^1^ Department of Neuromuscular Diseases UCL Queen Square Institute of Neurology London UK

**Keywords:** Guillain–Barré syndrome, history, pathogenesis, pathology, treatment

## Abstract

**Background and purpose:**

Since its description by Guillain, Barré, and Strohl in 1916, Guillain–Barré syndrome (GBS) has attracted a large literature. The author reviews the history of research into its pathogenesis and treatment to highlight promising avenues for future research.

**Methods:**

This is a nonsystematic personal review.

**Results:**

Since the early 1900s, the clinical picture of GBS has been illustrated in multiple series culminating in the ongoing International Guillain–Barré Syndrome study of 2000 patients. In the 1950s and 1960s, the inflammatory nature of the commonest form, acute inflammatory demyelinating polyradiculoneuropathy (AIDP), was described. In the 1990s, two axonal forms, acute motor–sensory axonal neuropathy and acute motor axonal neuropathy, were recognized. In the 1990s and early 2000s, these forms were shown to be due to antibodies against *Campylobacter jejuni* glycans cross‐reacting with glycolipids on axonal membranes. The pathogenesis of AIDP remains unknown, but T‐cell responses to the compact myelin proteins, P2 and P0, which cause experimental autoimmune neuritis, suggest that T cells are important. Randomized controlled trials in the 1970s and 1980s showed no benefit from corticosteroids. Trials in the 1980s showed benefit from plasma exchange and in the 1990s from intravenous immunoglobulin.

**Conclusions:**

Future research should seek biomarkers to identify subgroups with different treatment responses, define the true natural history of the disease with population‐based epidemiological studies, study the pathology in autopsies early in the disease, seek causative antibodies and confirm autoimmune T‐cell responses in AIDP, and expand treatment trials to include anti‐T‐cell agents.

## INTRODUCTION

Guillain–Barré syndrome (GBS) is one of the most striking neurological illnesses, capable of rendering a person completely paralyzed and ventilator‐dependent within a few days. Guillain, Barré, and Strohl recognized that the rapid onset of limb weakness with loss of tendon reflexes and a raised cerebrospinal fluid protein with a normal cell count constituted a discrete clinical syndrome. Subsequent clinical, neurophysiological, and pathological studies showed that the syndrome is heterogeneous. Increasingly refined pathological and immunological studies have investigated the pathogenesis of demyelinating and axonal subtypes. National and then international collaborations have accelerated progress in identifying triggering infections and defining and predicting the disease course. An international consensus group has agreed on management guidelines. Multicentre trials have identified effective treatments, and at last pharmaceutical companies are taking an interest. This review uses the history of research into GBS to propose avenues for future investigation.

## METHODS

This is a nonsystematic personal review.

## CLINICAL PICTURE

In 1916, Guillain, Barré, and Strohl distinguished GBS from other causes of acute ascending paralysis, emphasizing the absence of tendon reflexes and the high cerebrospinal fluid protein content and normal cell count [1]. The syndrome came to be regarded as an acute motor and sensory polyradiculoneuropathy predominantly affecting the limbs but often affecting the face, bulbar nerves, and respiration. In 1956, Miller Fisher drew attention to a syndrome of ophthalmoplegia, ataxia, and loss of the tendon reflexes, the Miller Fisher syndrome (MFS), which shared clinical features with GBS [2]. This is milder than GBS unless it develops into MFS‐GBS overlap syndrome. Other variants affecting different regions, such as the pharyngeal–cervical–brachial form, have been added over the years. The limits of the syndrome have been defined by a consensus statement and the European Academy of Neurology/Peripheral Nerve Society guideline [3, 4].

Further classification of GBS into subtypes has depended on deductions about the underlying pathology. In the early 1990s, McKhann and colleagues undertook clinicopathological studies of patients who died of GBS in Hebei Province, China and defined three forms of the disease [5]. The commonest form in Europe and North America is a demyelinating polyradiculoneuropathy that affects motor and sensory nerves, known as acute inflammatory demyelinating polyradiculoneuropathy (AIDP). Less common is an acute axonal form first described by Feasby and colleagues and called acute motor–sensory axonal neuropathy (AMSAN) by Griffin and colleagues [6]. A third form, called acute motor axonal neuropathy (AMAN), affects only motor axons [7]. The axonal forms may be severe or mild. In the severe form, there is axonal degeneration and prolonged disability. In the mild form, there is only conduction block attributed to a nodopathy and compatible with rapid recovery [8, 9].

The clinical picture has been documented in many series culminating in the ongoing International Guillain–Barré Syndrome (IGOS) under the leadership of Bart Jacobs in Rotterdam. This has collected the clinical records and blood samples from 2000 patients. Headline results from the first 1000 showed that the motor–sensory form was predominant in Europe/Americas (69%) and Asia (43%) but pure motor disease in Bangladesh (69%) [10]. The MFS and MFS‐GBS overlap syndromes occurred in 22% in Asia but only 11% in Europe and the Americas. The electrophysiologically defined axonal subtype was more common in Bangladesh (36%) than the other regions (6%). GBS can cause prolonged serious disability. After 1 year, 17% were unable to walk without aid in Europe/Americas, 31% in Bangladesh, and only 9% in Asia, where MFS, which is milder, was more common. Mortality was 5% in Europe/Americas, 2% in Asia, and 17% in Bangladesh, where treatment facilities were limited. The geographical variations could be caused by a mixture of differences in the incidence of precipitating factors or the prevalence of susceptibility genes. This international study has built a bank of clinical data and blood samples to help explore the disease course, precipitating agents, immune factors, and contributory genes. Such data from convenience samples are helpful, but they are biased towards people who have access to research centres. We need to look to formal population‐based epidemiological studies for data about true incidence and natural history. In a systematic review of 16 such studies collected from all over the world, the average annual incidence of GBS increased with age, from approximately 0.5 per 100,000 in children to approximately 2 per 100,000 in septuagenarians, being nearly 1.7 times greater in males than females [11]. A population‐based study in Denmark showed that IGOS was only capturing one third of the patients in that country, and these were on average those less severely affected [12].

## PATHOLOGY

Pathological studies have underpinned thinking about the pathogenesis of GBS. In 1955, Krücke gave the first clear description of the initial lesions consisting of multifocal mononuclear cell infiltration throughout the peripheral nervous system, especially at the junction of the ventral with the dorsal spinal roots [13]. In 1969, Asbury et al. confirmed this description and drew attention to the similarity with the pathology of experimental autoimmune neuritis (EAN) [14]. This description applied to the common motor and sensory form of the disease and gave rise to the name, AIDP. In severe cases, the intense inflammatory response causes axonal degeneration as well as demyelination. In the early 1990s, the already mentioned clinicopathological studies of Griffin and colleagues profoundly influenced thinking about different forms of GBS [5]. They obtained autopsies on three patients with AIDP in the first few days of the illness within 10 h after death. By immunohistochemistry, they showed deposition of complement components on the surface of Schwann cells in the affected ventral roots [15].

In 1986, Feasby et al. reported the autopsy of a patient with an axonal form of GBS; the clinical picture was typical of GBS, but the nerves were electrophysiologically inexcitable and the autopsy showed only axonal neuropathy, without demyelination or lymphocytic infiltration [6]. In the series of patients from China just mentioned, Griffin and colleagues described autopsies from similar patients in which the main pathological change was axonal degeneration with minimal inflammation and demyelination. They called this AMSAN. A striking ultrastructural feature was periaxonal and sometimes axonal infiltration of macrophages, a phenomenon that differs from AIDP but also occurs in rabies, which was the diagnosis in one of their patients [16, 17].

Griffin and colleagues distinguished a third type of GBS, AMAN, affecting motor nerve fibres and largely sparing sensory fibres [5, 7, 18]. In these cases, they identified immunohistochemically labelled macrophages penetrating the nodes of Ranvier and deposition of complement component C3d and immunoglobulin on the nodal axolemma and in the periaxonal space.

Peripheral nerve biopsies in AIDP have shown ultrastructural details of the process of demyelination with macrophage processes insinuating intact myelin sheaths at the apposed external surfaces of the myelin wraps, stripping, digesting, and eventually removing the myelin [19]. This is like the appearances in EAN [20]. Koike et al. recently showed in GBS biopsies that macrophages invade both at the internodes via the Schmidt–Lanterman incisures and at the paranodes separating the terminal Schwann cell loops from the axon [21]. In none of the biopsy studies has there been a report of ultrastructural change in the myelin in the absence of macrophage infiltration. These findings imply that demyelination is not directly caused by antibodies alone but is macrophage mediated. They do not exclude a role for T cells, which play the principal role in EAN. Like Griffin in autopsy material [15], Koike identified deposition of complement components along the myelin sheaths and at the paranodes, consistent with a role for antibody and complement [21].

Biopsies are rarely needed for diagnosis and the sural nerve usually selected for biopsy is sensory, distal, and remote from the sites of principal pathology in the ventral spinal roots and nerve trunks. Past advances have arisen more from autopsies than biopsies. Future research should study the principal sites of pathology postmortem in those rare patients who still sadly die in the early stages of the disease. In such an autopsy, Berciano illustrated an additional component to the pathology, namely axonal degeneration and oedema in the ventral roots. There, nerve fibres are potentially compressed by the tight layer of dura mater as they exit the dural sac, where increased endoneurial pressure could contribute to nerve damage [22].

## PATHOGENESIS

Two thirds of patients with GBS have had a recent infection. Almost every known infection has been implicated at one time or another. However, the only infections established as significantly associated by case–control studies are *Campylobacter jejuni*, cytomegalovirus, Epstein–Barr virus, *Mycoplasma pneumoniae*, Zika virus, hepatitis E, and *Haemophilus influenzae* [23]. All these infections, except *Haemophilus influenzae*, were documented in at least some patients in the IGOS study [24]. Despite claims to the contrary, there is no, or only a very small, increase in risk of GBS after SARS‐CoV‐2 [25].

The favoured view is that infections generate an immune response, especially antibodies, against antigens that are shared by glycolipids or proteins on Schwann cells, myelin, or axons. This has been convincingly established for AMAN following infection with *Campylobacter jejuni*, which is associated with antibodies to gangliosides, especially gangliosides GM1 and GD1a. Glycans resembling these gangliosides are present in the lipopolysaccharide in the *Campylobacter* membrane and generate an immune response that cross‐reacts with gangliosides present on human nerves. In 2001, Yuki reported a model of AMAN created by injecting a ganglioside mixture into rabbits [26]. The rabbits developed antibodies to ganglioside GM1 and paralysis due to an axonal neuropathy in which there was little inflammation but deposition of immunoglobulin on the axons and invasion of macrophages into the periaxonal space [27]. Soon afterwards, Willison created elegant mouse models that nicely demonstrate the mechanisms of nerve damage. In a model of AMAN, antibodies to ganglioside GD1a attach to, disrupt, and block conduction at the nodes of Ranvier in motor nerve fibres in a complement‐dependent fashion [28]. In a model of MFS, in which almost all patients have antibodies to ganglioside GQ1b, complement‐fixing antibodies to GQ1b engage and damage either the terminal motor axon or the terminal perisynaptic Schwann cell or both, depending on their fine specificity [29].

The pathogenesis of AIDP is much less clear. By comparison with axonal GBS, few patients with AIDP have antibodies to single gangliosides, although Rinaldi and colleagues have shown the proportion of patients with positive results is greatly increased from 11.8% to 62.4% when the gangliosides are presented in heterodimeric complexes [30]. Although similar antibodies are also present in normal and neurological disease control sera, they are less common. The role of antibodies against ganglioside complexes is unknown, nor is it known whether they are the consequence of the disease or critical for its pathogenesis. Their existence increases the complexity of the search for antibodies in AIDP and other forms of GBS.

By contrast, the search for antibodies to neural antigens other than gangliosides in AIDP has been tantalizingly unsuccessful. The simplest method for detecting antibodies is to apply sera to sections of peripheral nerve on slides, but even normal sera cause binding of immunoglobulin, which masks detection of specific antibodies. With monkey nerve as the substrate, Lleixa et al. detected strong binding to Schwann cells in 10% of 100 GBS sera and 1.8% of control sera [31]. Such reactivity is only present in a minority of cases, and the identity of the target antigen is unknown. Attempts to identify antibodies to compact myelin proteins have been negative [32, 33]. Searches for antibodies to nodal or paranodal proteins have given contradictory results. Devaux et al. found antibodies in sera from 28% of 100 patients and 4% of controls using human embryonic kidney cells transfected with NF186, gliomedin, NrCAM, or contactin [34], but Lleixa et al. found none [31]. However, one result stands out. Rinaldi and colleagues identified eight patients with antibodies to paranodal antigens with a stereotyped clinical picture of severe acute polyradiculoneuropathy and IgG1 antibodies to both the nodal (NF186) and paranodal (NF155) forms of neurofascin (panneurofascin antibodies) [35]. The neurophysiological studies of these patients were interpreted as showing nodal pathology or axonal degeneration rather than demyelination. The patients responded poorly to standard treatments but did improve after rituximab. The European Academy of Neurology/Peripheral Nerve Society guideline recommends testing patients with very severe GBS for antibodies against nodal–paranodal antigens [4].

Further searches for antibodies to Schwann cell and axonal antigens in untreated patients with GBS at an early stage of the disease would be worthwhile in the hope of identifying other subgroups with distinct clinical features and treatment responses. Such a search has been rewarding in chronic inflammatory demyelinating polyradiculoneuropathy, in which a subgroup of patients identified by IgG4 antibodies to paranodal antigens have characteristic clinical features, unresponsiveness to standard treatments, and improvement after rituximab [36, 37]. An international task force has classified these patients separately from chronic inflammatory demyelinating polyradiculoneuropathy as “autoimmune nodopathies” [38]. The potential role of antibodies to neural antigens in identifying subgroups of GBS has already been demonstrated by the tight association between MFS and antibodies to GQ1b [39]. The methods used for identifying antibodies are critical. Cell‐based assays for paranodal proteins have been more successful than enzyme‐linked immunosorbent assays, presumably because the protein is presented in its native configuration [35]. A myelinating human Schwann cell culture would be the ideal substrate for finding antibodies to antigens on myelin or Schwann cells, but such a model is lacking, and its development should be a research priority.

Because antibodies to Schwann cell and myelin antigens are rare and difficult to find, the old question arises whether AIDP is driven by T cells, and a recent paper renews interest in this idea. The histological appearances of AIDP closely resemble those of EAN, which is driven by a T‐cell immune response to compact myelin proteins, especially P2 and P0, not antibodies [40, 41]. There are even spontaneous forms of T‐cell‐driven autoimmune neuritis that develop in immunodeficient mouse strains and can be transferred by P0 T‐cell lines [42]. Initial attempts to identify T‐cell responses to myelin antigens in GBS were unsuccessful [33, 43]. However, Sukenikova and colleagues have now used state of the art techniques to identify CD4 T cells reacting with P2, P0, or PMP22 in the blood of 12 of 15 patients with AIDP but not, or rarely, in AMAN or Charcot–Marie–Tooth disease, and only in two of 21 healthy controls [44]. The initial responses were predominantly against P2 and to a lesser extent P0 and PMP22. Autoreactive single T‐cell clones had Th1‐like genes, a cytotoxic phenotype, and genes characteristic of autoimmunity. Furthermore, similar cells were present during the recovery as well as the acute stages of disease and in the cerebrospinal fluid of three patients and the sural nerve of the one tested. The techniques required to detect these responses are complex, expensive, and time‐consuming and will be difficult to replicate. Questions could be asked about the small numbers of patients and controls. The results fundamentally challenge current thinking about pathogenesis and would repay replication and further investigation. An immune response to an autoantigen could well involve both T cells and B cells, so that it might be an oversimplification to disregard either.

The question arises whether patients who develop GBS are genetically more susceptible than the general population. There are rare reports of the familial occurrence of GBS in sibling pairs or in successive generations [45]. Because the lifetime incidence of GBS is approximately 1 in 1000, these reports could be a coincidence. Many searches for genes associated with GBS have been undertaken in small studies. In a meta‐analysis, specific FcγR IIA, TNF‐α, TLR4, and HLA DRB genes, genes involving B‐cell, T‐cell, and innate immunity pathways, were more common in GBS than controls in Asian, European, or both populations [46]. Genome‐wide association studies on larger populations such as that available in IGOS would be worthwhile to refine these results and look for differences in associations between GBS subtypes.

## TREATMENT

That spontaneous improvement from GBS is the rule is a blessing for the patient but a disadvantage to the investigator trying to discover effective treatments. The Rotterdam group pioneered a prognostic scoring system that allows prediction of future outcome in individual patients with reasonable accuracy and have refined and recalibrated this with the IGOS database [47]. Significant adverse prognostic factors include severity of weakness, older age, and previous diarrhoea. Future research should examine whether the addition of other items, especially disease subtypes and antibodies to neural antigens, would increase the precision of the prognostic algorithm.

Corticosteroids were used for many years with claims and counterclaims about their efficacy. It was only when they were tested in large national and then international double‐blind randomized controlled trials that it became accepted that their apparent efficacy is illusory [48]. By contrast, plasma exchange is effective. In the 1980s, a North American trial with 245 participants showed that it reduced the time to walk unaided by >1 month (from a median of 85 days to a median of 53 days) and halved the time on the ventilator for ventilated patients (from a median of 48 days to 24 days) compared with supportive treatment [49]. This conclusion was confirmed by large French trials and endorsed by a Cochrane review [50]. Although not validated in a placebo‐controlled trial because of the ethical difficulty of performing sham exchange in critically ill patients, plasma exchange has become accepted worldwide as a first‐line treatment for GBS [4]. Small volume plasma exchanges are being investigated as an alternative to standard plasma exchange in resource‐poor regions of the world [51]. Because plasma exchange had become accepted as the standard treatment, new treatments had to be compared against it. A Dutch trial found that a single course of intravenous immunoglobulin (IVIg) was at least as efficacious as plasma exchange, a result that was replicated in other trials and endorsed in a Cochrane review [52, 53]. Consequently, IVIg is also accepted as a first‐line treatment [4]. Giving a second IVIg course was no more effective and was associated with more severe adverse events [54]. None of the other treatments tried so far has been shown to have a significant effect [55].

Randomized controlled trials are difficult in GBS because of its rarity, heterogeneity, and variable course. Consequently, large sample sizes are needed to detect a significant effect. In addition, there is the ethical imperative to give one of the standard treatments to both treatment groups and look for an additive effect from a potential new treatment. The requirement for large numbers in a conventional randomized controlled trial requires multinational multicentre studies. Despite these difficulties, the pharmaceutical industry has at last been attracted to the field. There are planned or ongoing trials of complement inhibitors, imlifidase, an IgG‐cleaving enzyme purified from *Streptococcus* pyogenes, and efgartigimod alfa, a neonatal Fc receptor blocker, which reduce IgG levels [56, 57]. These treatments depend on the underlying pathogenesis of GBS being IgG and complement dependent. If the findings of T‐cell responses in early GBS are confirmed, treatment with one of the many available anti‐T‐cell drugs should be investigated.

Randomized clinical trials with contemporary controls are difficult in GBS, because many important baseline variables need to be taken into account in statistical analyses [58], and new methods of testing novel treatments are needed. Although we will continue to rely on conventional placebo‐controlled trials for registering new treatments, screening trials analysed with Bayesian statistics and predefined efficacy thresholds based on historical databases, such as IGOS, could be used to select drugs, doses, and target participant groups. Ideally, a distinction would be made between axonal and demyelinating subtypes at the time of randomization, but this is complicated in that the electrophysiological subtype may change during follow‐up and require serial studies for confirmation. At present, electrophysiological subtype is not considered to influence the choice of treatment [4], but this conclusion is based on the absence, not presence, of evidence. Future trials need to identify different subgroups at the time of randomization and look for differences in response between them. The best method for classification of subtype needs to be investigated further. Trials need to have large enough samples to allow comparison of efficacy in each subtype. It is to be hoped that antibodies or other biomarkers specific for AIDP will be discovered and prove useful in selecting appropriate treatment, as suggested by the encouraging results with rituximab in patients with severe GBS and panneurofascin antibodies [35].

Finally, we must always remember that lying in the bed is a person who is weak, tired, often in pain, and very frightened. The use of immunotherapy and trials of modern drugs should be centred around expert nursing, medical, and holistic care throughout the illness.

## CONCLUSIONS

- Clinical and neurophysiological studies have divided GBS into demyelinating and axonal forms, the latter further separated into those with axonal degeneration and those with conduction block due to nodopathy. Further subdivision has begun with the discovery of patients with panneurofascin antibodies who are severely affected, do not respond to IVIg, but do respond to rituximab in small series. This response should be confirmed in formal trials. Other antibodies and biomarkers should be sought to define other subgroups with different prognoses and treatment responses.
- The large international study, IGOS, is valuable in describing the geographical variation of the disease, the infections that precipitate it, and the basis for developing more accurate prognostic algorithms. There is still a need for large population‐based studies to clarify the natural history of the disease subtypes.
- Autopsy studies have been key to understanding the pathogenesis of the demyelinating (AIDP) and axonal (AMSAN and AMAN) subtypes. Biopsies have illustrated the fine structure of the pathology of these subtypes. Early autopsies continue to be valuable in defining lesion distribution and identifying particularly vulnerable sites.
- *Campylobacter* infection has been shown to cause axonal subtypes by generating antibodies to gangliosides that target macrophages to destroy axons or block conduction at the nodes. Further research should seek antibodies responsible for AIDP and confirm the presence of T‐cell autoimmunity against the compact myelin proteins P2 and P0, which induce EAN in rodent models.
- Although plasma exchange and IVIg have been shown to be effective, death and severe residual disability due to axonal degeneration are too common even in treated patients. Trials of blocking the complement pathway or antibody elimination should continue. If the presence of T‐cell autoimmunity to myelin protein antigens is confirmed, inhibition of the T‐cell pathway with one of the many available anti‐T‐cell drugs should be explored. Candidate drugs could be screened in small samples compared with propensity‐matched historical controls from large databases to select candidates for large scale randomized trials with contemporary controls.

CONFLICT OF INTEREST STATEMENT

The author has no conflict of interest to disclose.

## Data Availability

Data sharing not applicable to this article as no datasets were generated or analysed during the current study.
